# Access to psychological therapies amongst patients with a mental health diagnosis in primary care: a data linkage study

**DOI:** 10.1007/s00127-024-02787-y

**Published:** 2024-11-06

**Authors:** Raquel Catalao, Matthew Broadbent, Mark Ashworth, Jayati Das-Munshi, Stephani L. Hatch, Matthew Hotopf, Sarah Dorrington

**Affiliations:** 1 Department of Psychological Medicine, Institute of Psychiatry, Psychology & Neuroscience, King’s College, London, UK; 2 South West London and St George´s Mental Health NHS Trust, London, UK; 3 NIHR Maudsley Biomedical Research Centre, South London and Maudsley NHS Foundation Trust, London, UK; 4 School of Population Health and Environmental Sciences, King’s College London, London, UK; 5 ESRC Centre for Society and Mental Health, King’s College London, London, UK

**Keywords:** Psychological therapies, Inequalities, Ethnicity, Population health

## Abstract

**Purpose:**

Significant numbers of people in England have fallen into a gap between primary care psychological therapies and specialist mental health services. We aim to examine pathways to care by looking at demographic variation in detection and referral to primary and secondary psychological services in south London.

**Methods:**

Longitudinal descriptive study using a record linkage between a primary care database (Lambeth DataNet) and a secondary care mental health database (CRIS). We extracted data on mental health diagnosis, prescriptions and episodes of care in mental health services for all patients of working age registered from 1 January 2008 to 1 March 2018 (pre-covid era).

**Results:**

Of those with a mental disorder detected in primary care (*n* = 110,419; 26.8%); 33.7% (*n* = 37,253) received no treatment; 21.3% (*n* = 23,548) exclusively accessed psychological treatment within NHS Talking Therapies and 7.6% accessed secondary care psychological therapies. People from minoritised groups were more likely to be prescribed psychotropic medication as the only treatment offered compared to the White British group. Men, Black African and Asian groups were less likely to access NHS Talking Therapies. People with a personality disorder diagnosis had the highest prevalence and number of NHS Talking Therapies treatment episodes (48.0%, *n* = 960), a similar percentage (44.1%, *n* = 881) received secondary care psychology treatment.

**Conclusion:**

Our study highlights marked inequalities in access to psychological therapies for men and people from some minoritised ethnic groups across primary and secondary care and how individuals with personality disorders are offered multiple short-term courses in NHS Talking Therapies even where this is not recommended treatment.

**Supplementary Information:**

The online version contains supplementary material available at 10.1007/s00127-024-02787-y.

## Introduction

An estimated 25% of people experience a mental health disorder every year, however only half of these are detected in primary care [1, 2]. Once a mental health disorder has been detected, prompt access to mental health care has been found to improve social, educational, occupational, physical health and mental health outcomes [3–5]. The UK National Institute for Health and Care Clinical Excellence (NICE) guidelines include access to a wide variety of therapies according to diagnostic complexity, from brief psychological interventions for people with common mental disorders [6] to long term psychodynamic therapy in secondary mental health services for patients with more complex emotional needs [7]. However, access to mental health services for people with mental health disorders is not equally distributed [8–10].

NHS Talking Therapies (formerly known as Improving Access to Psychological Therapies, IAPT) was introduced to standardise and improve access to primary care level psychological therapies in England. The Government set out an ambition for at least 25% of people (1.5 million) with common mental health conditions to access psychological therapies through NHS Talking Therapies each year by 2021. More recently, the NHS Long Term Plan proposes for an additional 380,000 adults and older adults to be able to access NICE approved NHS Talking Therapies by 2023/24, with a focus on people with long-term physical health conditions and medically unexplained symptoms [11]. Evidence shows that access to psychological services varies by a number of characteristics including age, ethnicity, disability and language [12]. Users from minoritized racial and ethnic groups experience inequalities in access to and experience of NHS Talking Therapies due to barriers at different pathway stages including referral and assessment [13, 14].

Furthermore, demand for mental health support in primary care is increasing and one of the biggest gaps reported by GPs is the increasing number of people who do not fit a clear referral pathway because of the complexity of their needs [15]. Part of this relates to growing levels of multimorbidity but there are also a wide variety of social factors – such as poverty, social isolation and trauma – and risk behaviours such as a drug and alcohol use and self-harm that can add to the complexity of a person’s needs and for which local NHS Talking Therapies services may be unable to offer appropriate services. Significant numbers of people in England have fallen into a gap between primary care psychological therapies and specialist mental health services [15]. Whereas there has been substantial investment in the NHS Talking Therapies, secondary care psychological therapies have struggled with insecure funding [16].

Diagnoses which are recognised in the NICE guidelines as needing NHS secondary care interventions, including longer-term therapies, include ‘severe mental illness’, ‘complex emotional needs’ and ‘personality disorder’. Unfortunately, the complexity and chronicity of these diagnoses often lead to barriers to care, including poor treatment, stigma, fragmented services and a lack of access to evidence based specialist services [17–19]. As a result, people with these diagnoses are frequently turned away from NHS Talking Therapies and also from secondary care, if they do not meet the stringent diagnostic criteria [18].

To gain a clearer understanding of the nature of variations in detection and access to services, it is necessary to take a whole population approach, which requires record linkage between primary, secondary mental health and NHS Talking Therapies health records, which to our knowledge has not yet been conducted.

### Aims

We aim to describe pathways to care by looking at demographic variation in detection of mental disorders in primary care and referral to primary and secondary psychological services in south London, using a primary care database linked with records from the local mental health service. We hypothesise firstly that there will be variation in identification of mental disorders in primary care and secondly in access to NHS Talking Therapies and secondary care psychological services by sex, ethnicity, neighbourhood deprivation and diagnosis.

## Methods

### Setting

The London Borough of Lambeth contains a relatively young and ethnically diverse population of 317,300 people, including large Portuguese, South American and Black populations. It has high levels of deprivation and population turnover [20]. Around 6% of the population speak a main language other than English and 41% were born outside of the UK [21].

### Data sources

#### Primary care source data: Lambeth DataNet (LDN)

Anonymised primary care data were extracted from the computerised medical records of all general practices in the London Borough of Lambeth (*n* = 48) as part of Lambeth DataNet (LDN). This dataset includes data from all registered patients including diagnoses and prescriptions, stored as Read codes (a standard coded vocabulary for clinicians to record patient findings and procedures, in health and social care IT systems across UK [22]).

#### Secondary care source data: clinical record interactive search (CRIS)

Secondary care data came from the Clinical Record Interactive Search (CRIS) [23], a large regional dataset containing anonymised clinical electronic records from the South London and the Maudsley NHS Foundation Trust (SLaM), Lambeth’s mental health provider. The local NHS Talking Therapies service, which provides primary care psychological treatment for common mental disorders, is provided by SLaM alongside secondary care services and is accessible through CRIS.

### Study design and data linkage

This is a longitudinal descriptive study using a record linkage between a primary care database and a secondary care mental health database in Lambeth. We analysed the records of patients registered from 1 January 2008 to 1 March 2018 (pre-COVID era). LDN data were extracted in December 2021 from the primary care clinical record system, EMIS Web. We restricted the sample to working age adults (aged 18–55 years at window start) who had at least a two-year registration in LDN during the study window. CRIS and LDN data were linked and stored by the SLaM Clinical Data Linkage Service, which provides a safe haven environment with strict governance arrangements. Data were linked using encrypted NHS numbers, which were subsequently removed and destroyed such that the linked dataset became fully anonymised.

### Measures

#### Demographic variables (LDN)

Information on individuals’ sex, year of birth, ethnicity and Index of Multiple Deprivation for area of residence were extracted from LDN. Index of Multiple Deprivation (IMD) is a national measure of relative deprivation for small areas (covering an average population of approximately 1500 individuals) in England. IMD scores were divided into quintiles, based on deprivation scores in Lambeth in 2015 [24]. Ethnicity, defined by UK census categories, was coded using seven subcategories of self-identified ethnicity (White British; White Other; Black African; Black Caribbean; Black Other; Asian and Other which includes those who identify with multiple ethnicities).

#### Mental health diagnoses (LDN and CRIS)

Data extracted from LDN included Read Codes on diagnoses of depression; anxiety; eating disorders; personality disorders and drug and alcohol misuse. Severe mental illness (SMI) status (a diagnosis of schizophrenia, bipolar disorder or other non-organic psychosis) was extracted using the Quality and Outcomes Framework (QOF) 2009 criteria [25] which is an annual reward and incentive programme for all GP practices in England. QOFs require GPs to identify individuals with selected Long-Term Conditions (LTCs) and meet clinical targets for their management.

Data extracted from CRIS included ICD-10 diagnosis recorded at first and last mental health care episode.

#### Long-term health conditions (LDN)

Presence of long-term health conditions was assessed using QOF (version 38 2017–2018) data from LDN [26]. We included a count of long-term conditions in our analysis defined by 24 conditions of public health relevance to the borough [27].

#### Antidepressants and antipsychotic prescription (LDN)

Antidepressant and antipsychotic prescription were measured in LDN as binary variables indicating whether the patients had received a prescription for an antidepressant or antipsychotic since 2008. These included antidepressants and antipsychotics included in the British National Formulary. A set of codes was used to extract antidepressants and to exclude medication used for other conditions, such as low dose tricyclic medication used for neuropathic pain [28].

#### Psychological treatment in primary care (CRIS)

Contact with NHS Talking Therapies was extracted from the CRIS dataset. We derived two outcomes related to treatment access: (1) ever completed a talking therapy episode at any time from 2008 to 2020 (only individuals who reached discharge stage in their records were included, those whose records indicated drop out or referral refusal were excluded) and (2) number of completed talking therapies episodes of care.

### Psychological treatment in secondary mental health services (CRIS)

Contact with secondary mental healthcare was defined as any contact with secondary mental healthcare services, measured as ever having an episode of care with secondary care services. This could include many routes into care (including GP referral) and different mental health interventions delivered in secondary care.

Psychological treatment within secondary mental health services was identified via two routes including: natural language processing algorithm for episodes of psychology treatment with at least 3 appointments attended in space of 3 months via face-to-face, video or telephone and any formal therapy face-to-face event attended with a type of therapy specified. Psychological treatment as defined in our study therefore included sessions delivered by qualified and training psychologists as well as sessions of psychotherapy delivered by other professionals who are not psychologists by training but who offered a type of talking therapy.

We derived two outcomes: (1) any episode of formal psychology or psychotherapy in secondary mental health services and (2) type of formal psychology or psychotherapy offered.

Type of formal therapy offered in secondary care included Cognitive Behavioural Therapy (CBT), family therapy, art therapy and several other modalities. We grouped therapies which focus on interpersonal difficulties in a group called “relational informed therapies” which include mentalisation based therapy; interpersonal therapy; individual psychodynamic therapy; cognitive analytical therapy; and dialectical behavioural therapy.

### Data analysis

Descriptive statistics were used to show prevalence of mental disorders in primary care records and psychological treatments offered in primary and secondary care. Univariate and multivariate logistic regression models were used to test association between socio-demographic characteristics and mental health diagnoses in primary care, mutually adjusting for ethnicity, sex and IMD.

Six categories of service use were defined as outcomes in those identified with a mental disorder in primary care: (1) no treatment recorded in primary and secondary care; (2) pharmacological treatment only (antidepressant or antipsychotic prescription in primary care); (3) NHS Talking Therapies (those with a completed treatment episode and excluding those with further treatment in secondary care); (4) any contact with secondary care mental health services; (5) psychotherapy within secondary care mental health services and (6) relational informed therapy within secondary mental health services. We derived 5 binary variables from the outcomes above to compare categories of service use with category 1 (no treatment recorded) as reference group: comparison 1 (category 1 and category 2); comparison 2 (category 1 and category 3) and so forth. Logistic regression was performed to test for association between socio-demographic characteristics (sex, ethnicity and IMD) and the five variables of service use. A multivariate logistic regression model adjusted for age as continuous variable and specific mental health diagnoses aggregated in a multilevel categorical variable (common mental disorders; SMI; personality disorders; alcohol and substance misuse disorders; other) was also performed. We conducted a complete case analysis.

## Results

### Sample and missing data

**Diagram 1:**
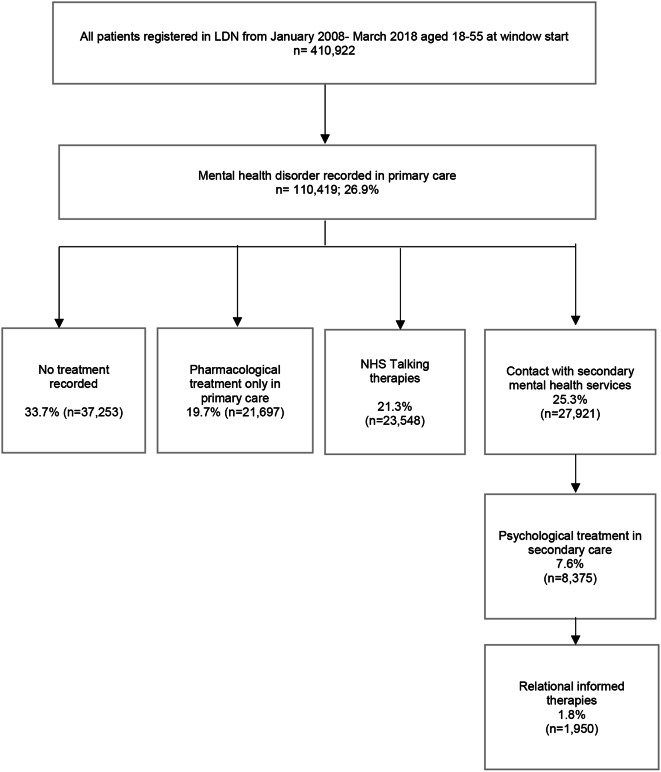
Flow diagram of patient sample

In our total primary care sample of 410,922 patients registered in LDN (diagram 1), median age at window start was 29 years (interquartile range 24–38). Men comprised 51.1% (*n* = 210,137) of the sample and women 48.9% (*n* = 200,785). There were no missing data for age and sex variables. Of the total sample, 3.4% had missing data on IMD for which median was 3 (interquartile range 2–5). Just over a third of the sample identified as White British (34.8%, *n* = 121,661); 31.6% as White Other (*n* = 110,523); 10.0% as Black African (*n* = 35,011); 5.5% as Black Caribbean (*n* = 19,182); 2.9% as Black Other (*n* = 10,194); 7.8% as Asian (*n* = 25, 278) and 8.0% identified as Other (*n* = 28, 124). We lacked data on ethnicity for 14.8% of total sample and a final sample of 350,105 patients with complete data were included in the adjusted analyses.

### Detection of mental health diagnoses in the primary care population

Over a quarter of the population (*n* = 110,419; 26.9%) had a mental health disorder recorded in primary care records (Table 1). Women were nearly twice as likely to be diagnosed with a mental health disorder compared with men. People living in areas of lower deprivation and those from minoritized ethnic groups were less likely to be diagnosed with mental health disorders in primary care compared to the White British group. People with two or more long term physical health conditions were over two times more likely to have a mental health diagnosis after adjustment.

**Table 1:** Characteristics of people with mental health diagnoses in primary care

Total sample (*n* = 410,922)	MH diagnoses (*n* = 110,419; 26.9% of total sample)	Association with MH diagnoses OR, 95%CI (*n* = 410, 922)	Association with MH diagnoses OR, 95%C mutually adjusted for age, sex, ethnicity (*n* = 350,105)
**Age (years)**			
**QUINTILES**			
**1 (18–24)** **2 (25–31)** **3 (32–38)** **4 (39–45)** **5 (46–53)**	33, 513 (30.4%) 23, 593 (21.4%) 20, 661 (18.7%) 17, 714 (16.0%) 14, 938 (13.5%)	1 (ref) 1.01 (0.99–1.03) 1.16 (1.14–1.18) *** 1.47 (1.44–1.51) *** 1.61 (1.58–1.65) ***	1 (ref) 1.06 (1.04–1.08) *** 1.38 (1.35–1.41) *** 1.87 (1.83–1.97) *** 2.03 (1.98–2.08) ***
**Sex**	**(*n* = 110**,**419)**	**(*n* = 410**,**922)**	**(*n* = 350**,**105)**
Female	63, 248 (57.3%)	1 (ref)	1 (ref)
Male	47 170 (42.7%)	0.63 (0.62–0.64) ***	0.63 (0.63–0.64) ***
**Ethnicity**	**(*n* = 101**,**146; 91**,**6%)**	**(*n* = 350**,**105)**	**(*n* = 350**,**105)**
White British	44, 171 (43.6%)	1 (ref)	1 (ref)
White Other	24 347 (24.2%)	0.50 (0.49–0.51) ***	0.48 (0.48–0.50) ***
Black African	8, 542 (8.4%)	0.57 (0.55–0.58) ***	0.49 (0.48–0.51) ***
Black Caribbean	7,791 (7.7%)	1.20 (1.16–1.24) ***	1.00 (0.96–1.03)
Black Other	3, 226 (3.2%)	0.81 (0.78–0.85) ***	0.72 (0.69–0.76) ***
Asian	5, 300 (5.2%)	0.47 (0.45–0.48) ***	0.44 (0.43–0.46) ***
Other	7,769 (7.7%)	0.66 (0.64–0.68) ***	0.63 (0.61–0.65) ***
**IMD**	**(*n* = 106**,**432; 96.4%)**	**(*n* = 396**,**950)**	**(*n* = 350**,**105)**
**QUINTILES**			
**1 (most deprived)** **2** **3** **4** **5 (least deprived)**	33,235 (31.2%) 43,518 (40.9%) 23, 790 (22.4%) 5,365 (5.0%) 524 (0.5%)	1 (ref) 0.93 (0.91–0.94) *** 0.89 (0.87–0.91) *** 0.92 (0.84–0.95) *** 0.87 (0.79–0.97) ***	1 (ref) 0.88 (0.86–0.90) *** 0.81 (0.80–0.83) *** 0.77 (0.75–0.80) *** 0.74 (0.65–0.82) ***
**Number of long-term physical conditions**	**(*n* = 110**,**419)**	**(*n* = 110**,**419)**	**(***n*** = 350**,**105)**
0	49, 786 (46.7%)	1(ref)	1(ref)
1	25,940 (23.5%)	2.18 (2.14–2.22) ***	1.79 (1.76–1.83) ***
2+	34,009 (30.8%)	3.14 (3.09–3.19) ***	2.46 (2.43–2.54) ***

*** *p*<0.001

### Service use amongst people with a mental health diagnosis detected

Of people with a recorded mental health diagnosis on their primary care record, 33.7% (*n* = 37,253) had no pharmacological or psychological treatment recorded in primary care or secondary care contact (diagram 1). Psychotropic medication (antidepressant or antipsychotic medication) was the only treatment recorded in 19.7% (*n* = 21,697) of people with a mental health diagnosis identified, whereas 21.3% (*n* = 23,548) had an episode of psychological treatment within NHS Talking Therapies. Approximately a quarter of people with a mental health diagnosis identified (25.3%; *n* = 27,921) were in contact with secondary mental health services and 7.6% (*n* = 8,375) accessed secondary care psychological therapies with 1.8% (*n* = 1,950) offered relational informed psychological therapies.

Men and those living in areas of least deprivation were more likely to have no treatment recorded after adjustment for age and mental health diagnoses (Table 2). They were less likely to be treated with medication or access NHS talking therapies or secondary care psychological therapies including relational informed therapies. People from all ethnic groups were more likely to be prescribed psychotropic medication in primary care as the only treatment offered, compared to White British, in both unadjusted and adjusted analyses (supplementary Tables 1 and 2). People from Black African and Asian groups were also less likely to access NHS talking therapies. People from Black African, Black Caribbean, Black Other and Other ethnicities and were more likely to have contact with secondary mental health services after adjustment for age and diagnosis. People from Black African, White Other and Asian ethnic groups were less likely to access psychological therapies in secondary care including relational informed therapies.

**Table 2:** Association between categories of service use and socio-demographic characteristics using “no treatment recorded” as reference group (adjusted model for age and mental health diagnoses)

No treatment recorded (reference group) (*n* = 37,253; 33.7%)	Pharmacological treatment in primary care only (*n* = 21,697; 19.7%) Adj OR (95%CI)	NHS talking therapies (*n* = 23,548; 21.3%) Adj OR (95%CI)	Contact with secondary mental health services (*n* = 27,921; 25.3%) Adj OR (95%CI)	Any secondary care psychological treatment (*n* = 8,375;7.6%) Adj OR (95%CI)	Secondary care relational informed treatment (*n* = 1,950; 1.8%) Adj OR (95%CI)
**Sex**	**(** ***n*** ** = 58,950)**	**(** ***n*** ** = 60,801)**	**(** ***n*** ** = 65, 174)**	**(** ***n*** ** = 45,628)**	**(** ***n*** ** = 39,203)**
Female	1(ref)	1(ref)	1(ref)	1(ref)	1(ref)
Male	0.82 (0.79–0.84) ***	0.71 (0.69–0.84) ***	0.81 (0.78–0.84) ***	0.69 (0.65–0.73) ***	0.48 (0.43–0.54) ***
**Ethnicity**	**(*n* = 53**,**282)**	**(*n* = 55**,**757)**	**(*n* = 59**,**400)**	**(*n* = 41**,**500)**	**(*n* = 35**,**494)**
White British	1(ref)	1(ref)	1(ref)	1(ref)	1(ref)
White Other	1.06 (1.01–1.11) *	0.96 (0.92–1.00)	0.94 (0.90–0.98) **	0.83 (0.77–0.90) ***	0.84 (0.74–0.96) *
Black African	1.11 (1.04–1.18) **	0.68 (0.63–0.73) ***	1.09 (1.02–1.16) *	0.95 (0.85–1.06)	0.66 (0.53–0.82) ***
Black Caribbean	1.17 (1.08–1.26) ***	1.34 (1.25–1.44) ***	1.72 (1.60–1.84) ***	1.41 (1.27–1.58) ***	1.33 (1.10–1.61) **
Black Other	1.28 (1.14–1.43) ***	1.22 (1.10–1.36) ***	1.79 (1.61–1.98) ***	1.66 (1.42–1.94) ***	1.30 (0.98–1.73)
Asian	1.22 (1.12–1.32) ***	0.87 (0.81–0.95) **	1.06 (0.98–1.16)	0.85 (0.73–0.98) *	0.72 (0.55–0.94) *
Other	1.16 (1.07–1.24) ***	1.16 (1.08–1.24) ***	1.52 (1.42–1.63) ***	1.39 (1.25–1.55) ***	1.25 (1.03–1.51) *
**IMD**	**(*n* = 56**,**719)**	**(*n* = 58**,**442)**	**(*n* = 62**,**739)**	**(*n* = 43**,**847)**	**(*n* = 37**,**613)**
1 most deprived	1(base)	1(base)	1(base)	1(base)	1(ref)
2	0.87 (0.83–0.91) ***	0.92 (0.88–0.96) ***	0.77 (0.74–0.80) ***	0.81 (0.76–0.86) ***	0.78 (0.69–0.87) ***
3	0.77 (0.74–0.81) ***	0.83 (0.79–0.87) ***	0.62 (0.59–0.65) ***	0.65 (0.60–0.70) ***	0.60 (0.52–0.69) ***
4	0.76 (0.70–0.82) ***	0.74 (0.69–0.81) ***	0.52 (0.48–0.57) ***	0.53 (0.46–0.62) ***	0.57 (0.44–0.73) ***
5 least deprived	0.96 (0.77–1.21)	0.68 (0.54–0.86) ***	0.47 (0.36–0.62) ***	0.40 (0.23–0.67) **	0.28 (0.09–0.88) *

*<0.05 **<0.005 *** <0.001

### Contact with talking therapies and secondary care psychological services for people with anxiety and depression

The prevalence of different mental health disorders in primary care is shown in Table 3. Approximately a quarter of the sample (24.7%) had a common mental disorder (CMD) diagnosis Anxiety and Depression were the most prevalent mental disorders in primary care (16.4% and 17.0% respectively), but less than half of patients diagnosed with CMDs had a Talking Therapies treatment episode (Table 3). Co-morbid depression and anxiety were frequent, with 50.7% of those with coded depression also had a code for anxiety.

**Table 3:** Mental disorders recorded in primary care and number (n) and percentage (%) offered psychological treatment in talking therapies and secondary care

Total (*n* = 410,922)	Diagnosed in primary care *n*, (%)	*n*, (%) with episode of care in Talking Therapies (*n* = 49,193; 12.0%)	Median number of Talking Therapies episodes [IQR]	*n*, (%) in contact with secondary care	*n*, (%) with episode of secondary care psychotherapy (*n* = 9,424; 2.3%)	*n*, (%) in contact with secondary care with congruent diagnosis in primary care
Eating disorders	2,366 (0.6%)	803 (33.9%)	1 [1–2]	937 (39.6%)	468 (19.8%)	457 (48.8%)
Anxiety	67,314 (16.4%)	25,435 (37.8%)	1 [1–2]	15,987 (23.8%)	5,033 (7.3%)	1,698 (10.6%)
Depression	69,733 (17.0%)	28,742 (41.2%)	1 [1–2]	20,866 (29.9%)	6,112 (8.8%)	4,265 (20.4%)
Drug and Alcohol misuse	15,489 (3.8%)	4,598 (29.7%)	2 [1–3]	7,995 (51.6%)	2,261 (14.6%)	1,272 (15.9%)
Personality Disorders	1,999 (0.5%)	960 (48.0%)	2 [1–3]	1,545 (77.3%)	881 (44.1%)	413 (26.3%)
Severe Mental Illness	6,427 (1.7%)	1,794 (27.9%)	2 [1–3]	5,747 (85.2%)	3,088 (48.1%)	3,270 (59.7%)
Antidepressant prescription	67,214 (16.4%)	24,415 (36.3%)	1 [1–2]	19,358 (28.8%)	5,761 (8.6%)	-
Antipsychotic prescription	6,661 (1.6%)	2,269 (34.1%)	2 [1–3]	5,740 (86.2%)	3, 246 (48.7%)	-
Any Mental Health diagnosis	131,277 (28.5%)	38,069 (32.1%)	1 [1–2]	28,281 (23.8%)	8,534 (7.2%)	-

The majority of people accessing NHS Talking Therapies completed one treatment episode (*n* = 29,287; 59.5%) and over half of people with a CMD accessing psychological therapy in primary care (54.9%; *n* = 20,009) completed only one treatment episode. About 20.6% of those with a CMD diagnosis completed 3 or more treatment episodes. People with drug and alcohol misuse, personality disorders and severe mental illness had higher number of multiple NHS Talking Therapies treatment episodes.

### Contact with talking therapies and secondary care psychological services for people with personality disorders

Whereas a small number of people had a record of personality disorders diagnosis in primary care (*n* = 1,999; 0.5% of the sample population), it was the mental health diagnosis with the highest prevalence of completed NHS Talking Therapies treatment episodes (48.0%, *n* = 960). A total of 44.1% (*n* = 881) of those with a personality disorder recorded in primary care received secondary mental health psychology treatment, although 77.3% were in contact with mental health services (Table 3).

There were no significant differences in contact with mental health services or in diagnosis of personality disorders between sexes (OR 0.94 95%CI (0.87–1.02) *p* = 0.13) but 57.4% of people diagnosed with a personality disorder in primary care were of White British ethnicity, people from ethnic minority groups were less likely to have a diagnosis of personality disorder (OR 0.87 95%CI (0.85–0.89) *p* < 0.001). Amongst people with a personality disorder diagnosis there was high co-morbidity: 80.2% (*n* = 1,604) had a coded co-diagnosis of depression. 68% of people (*n* = 1,360) with a personality disorder diagnosis were treated with antidepressants and 37.2% (*n* = 744) with antipsychotic medication. Of people with personality disorder receiving NHS Talking Therapies, 24.6% (*n* = 236) had two treatment episodes and 35.1% (*n* = 337) had three or more treatment episodes.

Of the total 1,999 people with a diagnosis of personality disorder in primary care, 413 (26.3%) had a personality disorder diagnosis in secondary care and half of those with a personality disorder recorded in secondary care (*n* = 799) were coded with same diagnosis in primary care.

### Contact with talking therapies and secondary care for people with SMI

People with SMI comprised 1.6% (*n* = 6,427) of the total sample and had the highest prevalence of contact with secondary mental health services (85.2% *n* = 5,747) and secondary care psychological treatment (48.1% *n* = 3,088) (Table 3). SMI was the diagnosis with most congruent recording in primary and secondary care (59.7% matching). Men (OR 1.26 95%CI (1.19–1.32) *p* < 0.001) and Black African (OR 1.21 95%CI (1.12–1.32) *p* < 0.001); Black Caribbean (OR 2.18 95%CI (2.01–2.37) *p* < 0.001); Black Other (2.26 95%CI (2.03–2.52) *p* < 0.001) and people from Other ethnicities (OR 1.12 95%CI (1.02–1.24) *p* = 0.018) were more likely to have an SMI diagnosis.

The vast majority of patients with SMI were treated with antipsychotic or antidepressant medication (78.5% *n* = 5,047); 27.9% (*n* = 1,794) completed a treatment episode with NHS Talking therapies.

The majority of patients with SMI accessing psychological treatment in secondary were offered CBT (65.0% *n* = 2,007), less than a quarter accessed family therapy (20.3% *n* = 629) and 15.6% accessed relational informed therapies.

### Types of psychological therapy offered in secondary mental health services

A total of 8,375 people had a type of formal psychotherapy recorded in their clinical notes. Of these 7,441 (88.8%) were identified via the algorithm for psychology appointments face to face with at least 3 appointments attended in a space of 3 months. Data were available on type of therapy offered in secondary care for 7,880 patients (94.1%) of the total with a formal psychotherapy episode recorded (Table 4). Of note patients could be offered more than one modality of treatment. The majority of therapy offered in secondary mental health services was Cognitive Behavioural Therapy (*n* = 4,806; 61.0%); 13.6% (*n* = 1,074) of patients accessed family therapy and less than 20% of patients access relational informed psychotherapies. Irrespective of treatment modality, the vast majority of patients completed fewer than ten sessions of psychological therapy.

**Table 4:** Types of psychological therapy offered in secondary care

Types of therapy offered	*N* (%) total = 7,880 therapy type recorded	Number of sessions completed	
Cognitive Behavioural Therapy	4,806 (61.0%)	1–5	2,014 (42.0%)
		6–10	1,251 (26.0%)
		11–20	819 (17.0%)
		> 20	722 (15.0%)
Family therapy	1,074 (13.6%)	1–5	719 (67.8%)
		6–10	74 (7.0%)
		11–20	102 (9.6%)
		> 20	166 (15.6%)
Art therapy	1,754 (22.2%)	1–5	1,082 (61.7%)
		6–10	243 (13.9%)
		11–20	228 (13.0%)
		> 20	201 (11.4%)
Group therapy	1,131 (14.3%)	1–5	742 (65.6%)
		6–10	127 (11.2%)
		11–20	79 /7.0%)
		> 20	183 (16.2%)
Individual Psychodynamic psychotherapy	1,303 (16.5%)	1–5	769 (59.0%)
		6–10	99 (7.6%)
		11–20	101 (7.8%)
		> 20	334 (25.6%)
Cognitive analytical therapy	585 (7.4%)	1–5	250 (42.7%)
		6–10	34 (7.8%)
		11–20	159 (27.2%)
		> 20	142 (24.3%)
Dialectical Behavioural Therapy	124 (1.6%)	1–5	91 (73.4%)
		6–10	10 (8.1%)
		11–20	7 (5.6%)
		> 20	16 (12.9%)
Interpersonal therapy	115 (1.5%)	1–5	96 (83.5%)
		6–10	3 (2.6%)
		11–20	6 (5.2%)
		> 20	10 (8.7%)
Mentalisation Based therapy	352 (4.5%)	1–5	163 (46.3%)
		6–10	49 (13.9%)
		11–20	39 (11.1%)
		> 20	101 (28.7%)
Eye Movement Desensitization and Reprocessing Therapy	74 (1.0%)	1–5	48 (64.9%)
		6–10	9 (12.2%)
		11–20	8 (10.8%)
		> 20	9 (12.2%)

## Discussion

In this local representative sample of primary care patients, we explored pathways into mental health care. Consistent with our hypothesis and previous studies [9, 10], there are variations in detection of mental disorders in primary care by sex, ethnicity and level of deprivation. There are also striking disparities in access to treatment and about a third of patients identified with a mental disorder have no treatment recorded. Similar to previous local and national reports [12–14], our results highlight inequalities in access to NHS Talking Therapies treatment for men and people from some minoritised ethnic groups. People from ethnic minority groups were less likely to have a mental health diagnosis in primary care but when diagnosed, were more likely to be treated with medication only. People from Black ethnic groups were more likely to be in contact with secondary mental health services and have a SMI diagnosis. Fewer than half of patients irrespective of mental health diagnosis, are able to access psychological therapies. This is despite surveys showing that patients have a strong preference for psychological treatments [29].

We found that these inequalities in access to NHS Talking Therapies are mirrored by inequalities in access to secondary care specialised psychotherapies particularly relational informed modalities. National data has previously shown that patients from minoritised groups with SMI in secondary care are less likely to be offered NICE guidance CBT and family therapies [30]. Men are more likely to die by suicide, and the higher rates of identified but not treated mental disorders in our study highlight a need to develop interventions to improve men´s help seeking behaviour and engagement with psychological therapies [31]. Similarly, interventions to improve detection of metal disorders in ethnic minority groups and develop services that meet the needs of the entire community require further investment and resources [13, 14].

Our study shows that more complex patients, particularly those with a personality disorder diagnosis, often receive multiple NHS Talking therapies treatment episodes with only a small percentage accessing psychological treatments in secondary mental health services. This is despite NICE guidance recommending first line treatment with specialised psychological therapies [7].

NHS Talking Therapies has expanded to include short term treatments for patients with more complex needs [32], without providing the long-term therapies previously provided by secondary mental healthcare services [14].The number of people with complex emotional needs identified, who do not respond to NHS Talking Therapies treatment, is increasing [33, 34]. Just under half of all referrals that completed Talking Therapies treatment in 2016-17 recovered [35]. With increased investment and promotion of primary care psychological services the number of patients likely to require further specialised therapies is expected to further rise. NHS Talking Therapies is meant to operate in a stepped care model approach but there is lack of clear pathways of care into more specialised psychological services for those patients who do not fully respond to step 4 (more intensive) treatment. There is currently no policy drive to improve access to secondary care psychological services. Our data shows that patients with more complex diagnoses make up a substantial part of repeat treatment episodes in NHS Talking Therapies. It echoes primary care reports of a large gap between NHS Talking Therapies and ability to access more specialised treatments if the patient is not judged “severe” or sufficiently “high-risk” to meet the rising thresholds of these stretched specialist services [15].

Our study also shows marked inconsistencies in recorded diagnoses between primary and secondary care with large implications for understanding local prevalence of mental health disorders and appropriately develop and commission services. People who are eligible for more specialised secondary care psychotherapy services, particularly those with personality disorders, continue to go undetected despite presenting across a range of medical and multi-agency settings [36, 37]. A personality disorder diagnosis was recorded in 0.5% of our sample, despite estimates of 24% prevalence in primary care attendees in the UK [37]. Less than half of patients with personality disorder detected in primary care accessed specialised care, most had other overlapping mental health diagnoses and were treated with medication, highlighting barriers to care even in those identified with more complex needs. Current NICE guidance clearly advocates that brief psychological treatments should not be offered for those with a personality disorder diagnosis and treatment of co-morbid depression and anxiety should take place within a well-structured programme [7].

As shown by our results, lack of detection by primary care and NHS Talking Therapies of more complex diagnoses can lead to ineffective low intensity psychological therapies offered, which may be inappropriate for patients with more complex needs [38], predicts poorer outcomes of treatment [39] and some argue can cause iatrogenic harm [40]. Most screening assessments done by NHS Talking Therapies are protocolised and conducted by psychological wellbeing practitioners who are trained to diagnose by screening for NHS Talking Therapies treated conditions [41]. The focus is on finding a problem descriptor that fits the service model, rather than assessing the complexity of the case. Despite a recent interest in trauma-informed care, the current system does not actively screen for trauma, personality disorders [42, 43], nor provide a developmental approach [40].

Even when more complex patients make it to secondary care services, our results highlight the strong emphasis in provision of CBT with only a small minority of patients being able to access other types of therapy. Less than a quarter of patients with SMI accessed family therapy in our sample, despite NICE guidance advocating for this treatment approach alongside CBT [44]. Provision of therapies using a relational approach including psychodynamic therapy was low.

### Strengths and limitations

To our knowledge this is one of the first studies investigating pathways to psychological therapies at population level using data linkage of health records across primary and secondary care. Despite the overall national investment in NHS Talking Therapies [32], research on pathways between primary and secondary care psychological therapies is limited. We extracted data on a large sample of representative adults of working age registered in primary care in the borough of Lambeth. Similar research in other areas of the country would be important for the generalisation of the results presented here although national reports highlighted that the gap between provision of psychological therapies for more complex patients is widespread [15]. NHS Digital publishes data on the number of patients accessing Talking Therapies across the country but it does not report the proportion of those with identified mental illness in primary care accessing treatment nor does it provide the number of those with repeated Talking Therapies episodes [45], further highlighting the pertinence of our study. We restricted our analysis to those of working age as NHS Talking Therapies were initially commissioned for this age group. Despite extension of services to all of those aged over 18 in 2010, those aged over 65 continue to be under-represented in services [46].

Diagnostic codes were derived from clinical codes added at clinicians’ discretion which represent detection of mental illness in primary and secondary care rather than the population prevalence of disease and the validity of specific diagnoses needs to be taken into consideration in view of the large inconsistencies recorded. The large variability in codes used in primary databases in the UK to detect mental health outcomes has been previously highlighted [47], and under detection of mental illness by primary care clinicians remains a widespread issue [6]. We focused our analysis on CMDs, personality disorders and SMI diagnoses but further studies should consider other mental disorders. We used broad groupings of minoritised ethnic groups, which do not capture within group variation and about 14% of the total sample lacked data on ethnicity, despite lower levels of missing data in those with identified mental disorders. Data on type of therapy offered in secondary care needs to be interpreted with caution as this can be inconsistently recorded by clinicians. As our study relies on data coded by clinicians, there may also be unknown missing data on several variables that could impact our results. We also lacked data on access to private talking therapies, which may be an important pathway to mental health care particularly for the least deprived patients.

## Conclusion

Our study highlights marked inequalities in access to psychological therapies for men and people from some ethnic minority groups across primary and secondary care and how complex patients are being offered multiple short-term courses in NHS Talking Therapies even for diagnoses where low intensity CBT is not recommended treatment. There is a bias in the system towards non complexity leading to high number of repeat short treatment episodes rather than provision of evidence-based care with potential implications for patient recovery as well as cost-effectiveness of the system.

## Electronic supplementary material

Below is the link to the electronic supplementary material.

Supplementary Material 1

## Data Availability

The data accessed by CRIS-LDN linkage remain within an NHS firewall and governance is provided by the CRIS Oversight Committee reporting to relevant information governance clinical leads and is therefore not publicly available.

## Declarations

### Patient and public involvement

CRIS and LDN were developed with extensive service user involvement and adheres to strict governance frameworks managed by service users. The Maudsley Biomedical Research Center data linkage service user and carer advisory group and Health Watch Lambeth provided feedback and advice to researchers on the project proposal.
