# Supplementary material for: Access to psychological therapies amongst patients with a mental health diagnosis in primary care: a data linkage study
Source: Soc Psychiatry Psychiatr Epidemiol. 2024 Nov 6;60(9):2149–61. doi: 10.1007/s00127-024-02787-y (PMC12378146; doi:10.1007/s00127-024-02787-y)
Supplement: Supplementary file 1 — Supplementary Material 1 [file 127_2024_2787_MOESM1_ESM.docx]

**Table S1. Association between categories of service use and socio-demographic characteristics with “no treatment recorded” as reference group (unadjusted model).**

| **No treatment recorded**  **(Reference group)**  **(n=37,253; 33.7%)** | **Pharmacological treatment in primary care only**  **(n=21,697; 19.7%)**  **OR (95%CI)** | **NHS talking therapies**  **(n=23,548; 21.3%)**  **OR (95%CI)** | **Contact with secondary mental health services**  **(n=27,921; 25.3%)**  **OR (95%CI)** | **Any secondary care psychological treatment**  **(n=8,375;7.6%)**  **OR (95%CI)** | **Secondary care relational informed treatment**  **(n=1,950; 1.8%)**  **OR (95%CI)** |
| --- | --- | --- | --- | --- | --- |
| **Sex** | **(n=58,950)** | **(n=60,801)** | **(n=65, 174)** | **(n=45,628)** | **(n=39,203)** |
| Female | 1(ref) | 1(ref) | 1(ref) | 1(ref) | 1(ref) |
| Male | 0.82 (0.79 – 0.84) *** | 0.66 (0.64 - 0.69) *** | 1.16 (1.12 - 1.19) *** | 1.07 (1.02 - 1.13) ** | 0.74 (0.68 - 0.82) *** |
| **Ethnicity** | **(n=53,282)** | **(n=55,757)** | **(n=59,400)** | **(n=41,500)** | **(n=35,494)** |
| White British | 1(ref) | 1(ref) | 1(ref) | 1(ref) | 1(ref) |
| White Other | 1.11 (1.06 - 1.16) *** | 0.98 (0.94 - 1.03) | 0.90 (0.87 - 0.94) *** | 0.78 (0.73 - 0.83) *** | 0.77 (0.68 - 0.87) *** |
| Black African | 1.42 (1.33 - 1.51) ** | 0.72 (0.67 - 0.77) *** | 1.17 (1.10 - 1.24) *** | 1.20 (1.09 - 1.31) *** | 0.63 (0.51 - 0.77) *** |
| Black Caribbean | 1.57 (1.46 - 1.69) *** | 1.35 (1.26 - 1.45) *** | 2.04 (1.92 – 2.18) *** | 2.03 (1.86 - 2.22) *** | 1.57 (1.32 - 1.86) *** |
| Black Other | 1.54 (1.38 - 1.72) *** | 1.24 (1.12 - 1.38) *** | 2.11 (1.92 – 2.32) *** | 2.36 (2.08 - 2.67) *** | 1.50 (1.16 - 1.94) ** |
| Asian | 1.38 (1.28 - 1.49) *** | 0.92 (0.85 - 0.99) * | 1.01 (0.94 - 1.09) | 0.90 (0.80 - 1.01) | 0.66 (0.51 - 0.85) ** |
| Other | 1.24 (1.15 - 1.33) *** | 1.19 (1.12 - 1.28) *** | 1.45 (1.36 - 1.54) *** | 1.40 (1.28 - 1.54) *** | 1.20 (1.01 - 1.43) * |
| **IMD** | **(n=56, 719)** | **(n=58,442)** | **(n=62, 739)** | **(n=43,847)** | **(n=37,613)** |
| 1 most deprived | 1(base) | 1(base) | 1(base) | 1(base) | 1(ref) |
| 2 | 0.81 (0.77 - 0.84) *** | 0.92 (0.88 - 0.96) *** | 0.74 (0.71 - 0.76) *** | 0.78 (0.74 - 0.83) *** | 0.76 (0.69 - 0.85) *** |
| 3 | 0.73 (0.69 - 0.77) *** | 0.83 (0.80 - 0.87) *** | 0.57 (0.55 - 0.60) *** | 0.60 (0.56 - 0.64) *** | 0.58 (0.51 - 0.67) *** |
| 4 | 0.71 (0.65 - 0.77) *** | 0.76 (0.70 - 0.82) *** | 0.45 (0.42 - 0.49) *** | 0.45 (0.39 - 0.51) *** | 0.52 (0.41 - 0.66) *** |
| 5 least deprived | 0.83 (0.66 - 1.03) | 0.71 (0.56 - 0.90) ** | 0.35 (0.27 - 0.46) *** | 0.27 (0.16 - 0.44) ** | 0.20 (0.06 - 0.64) * |

^*<0.05 **<0.005 *** <0.001^
